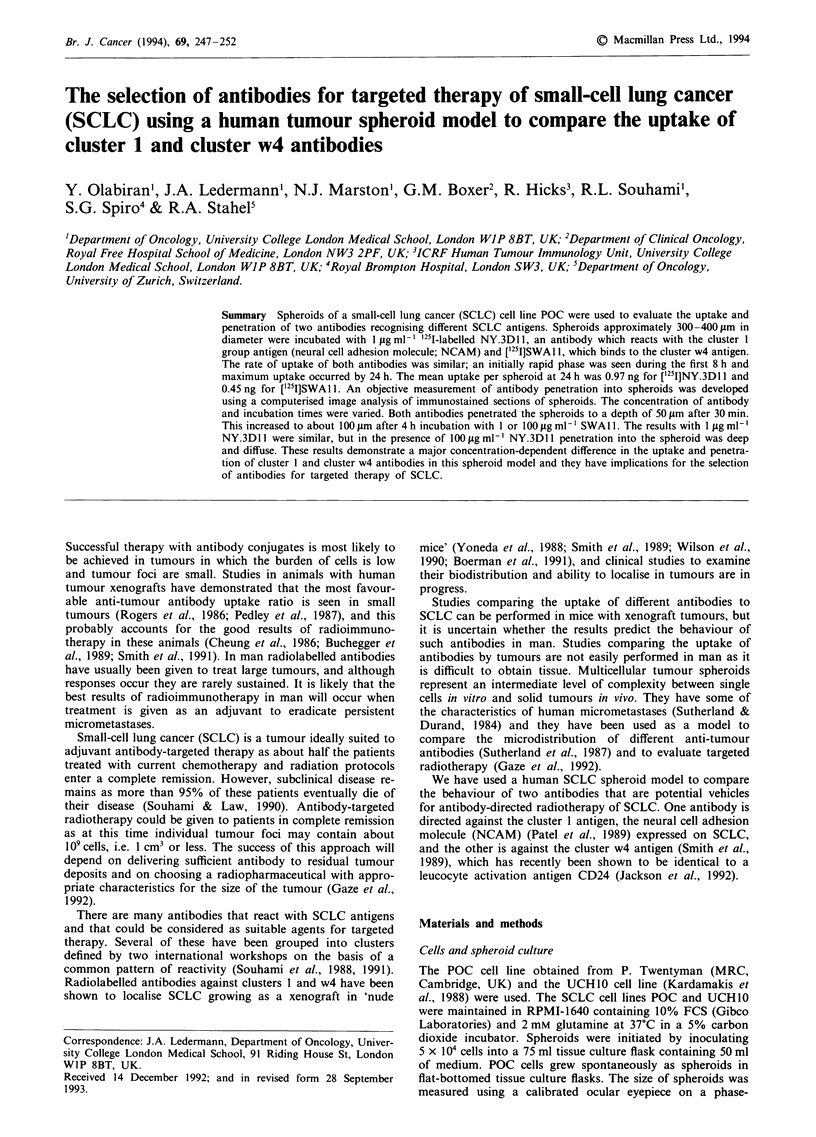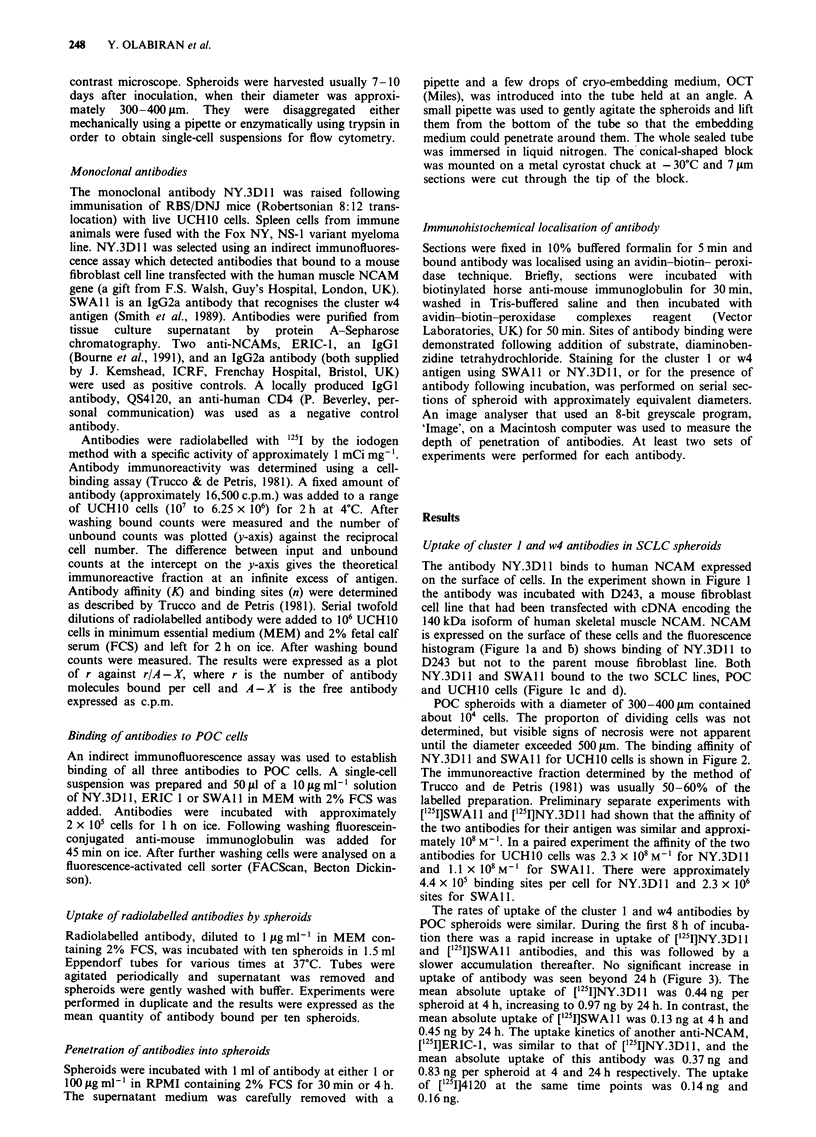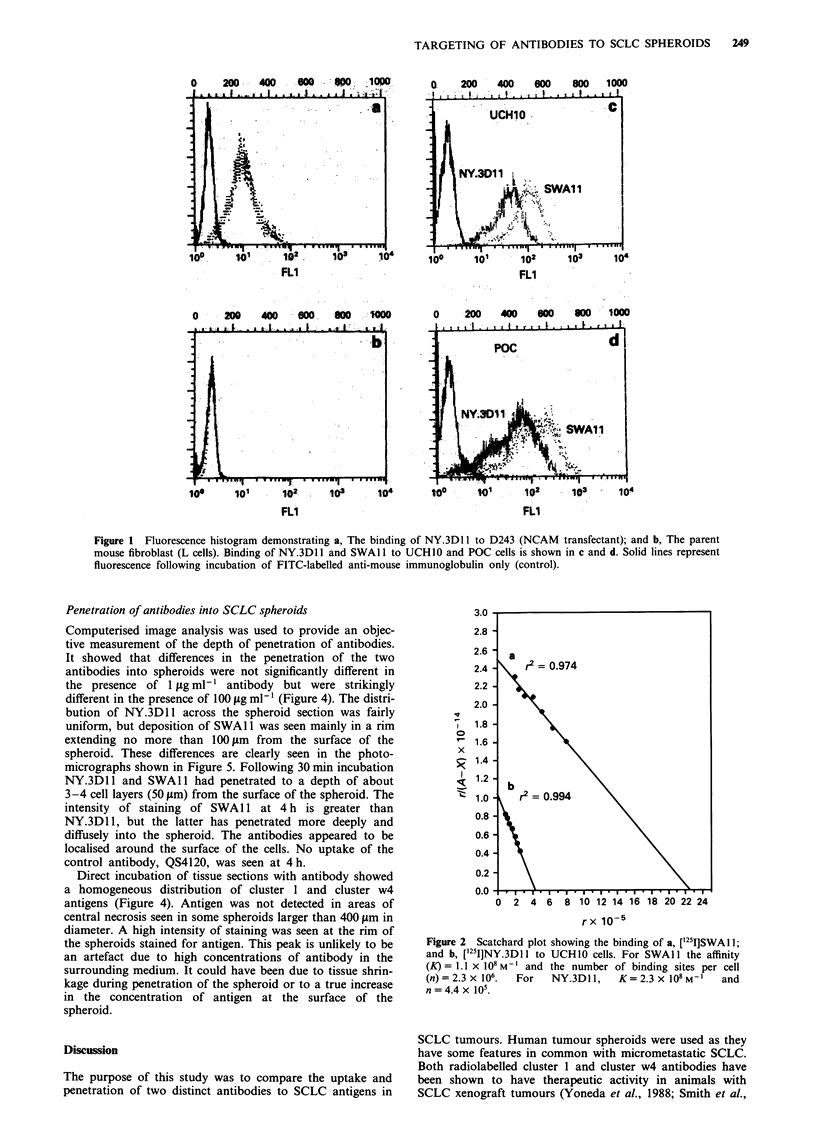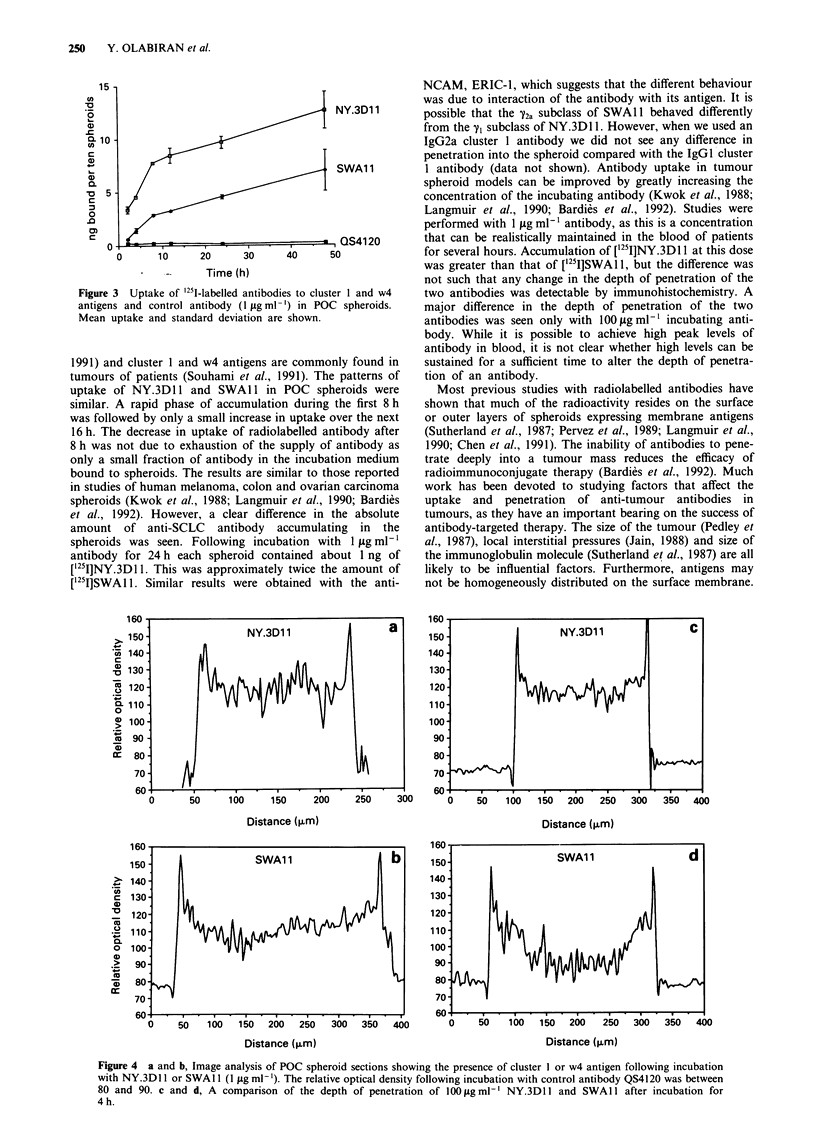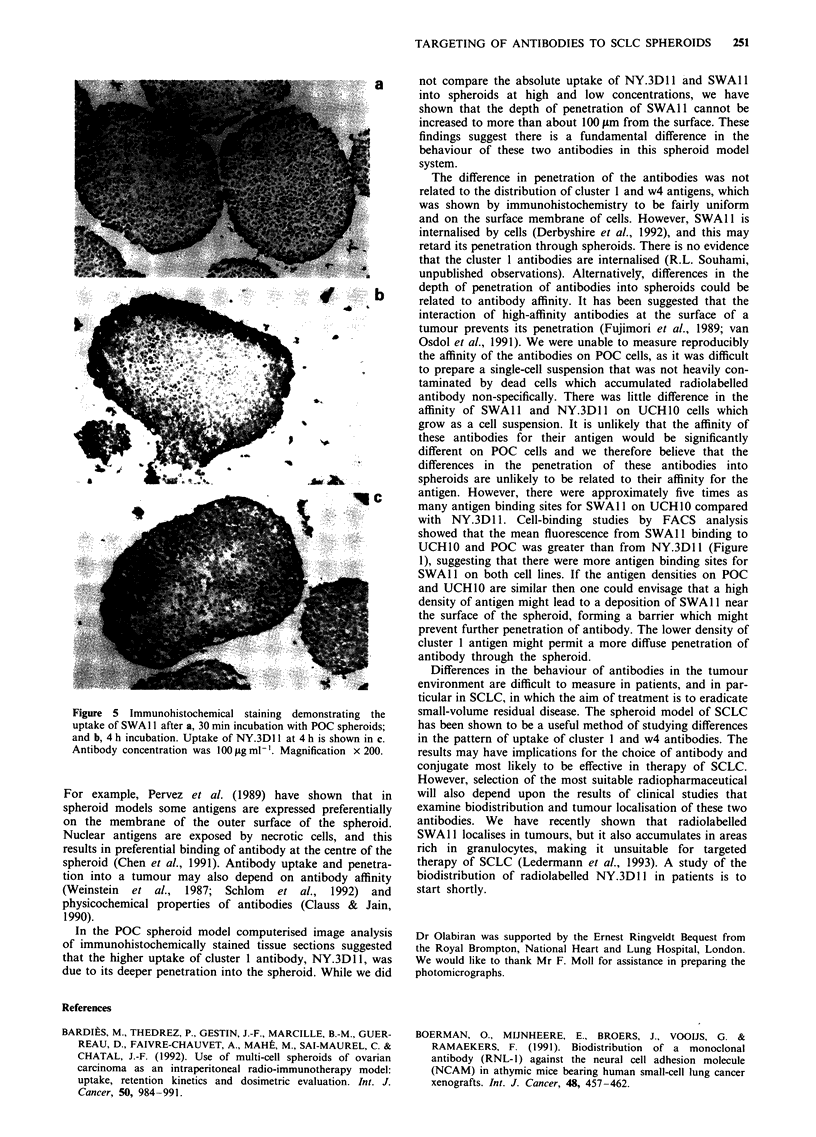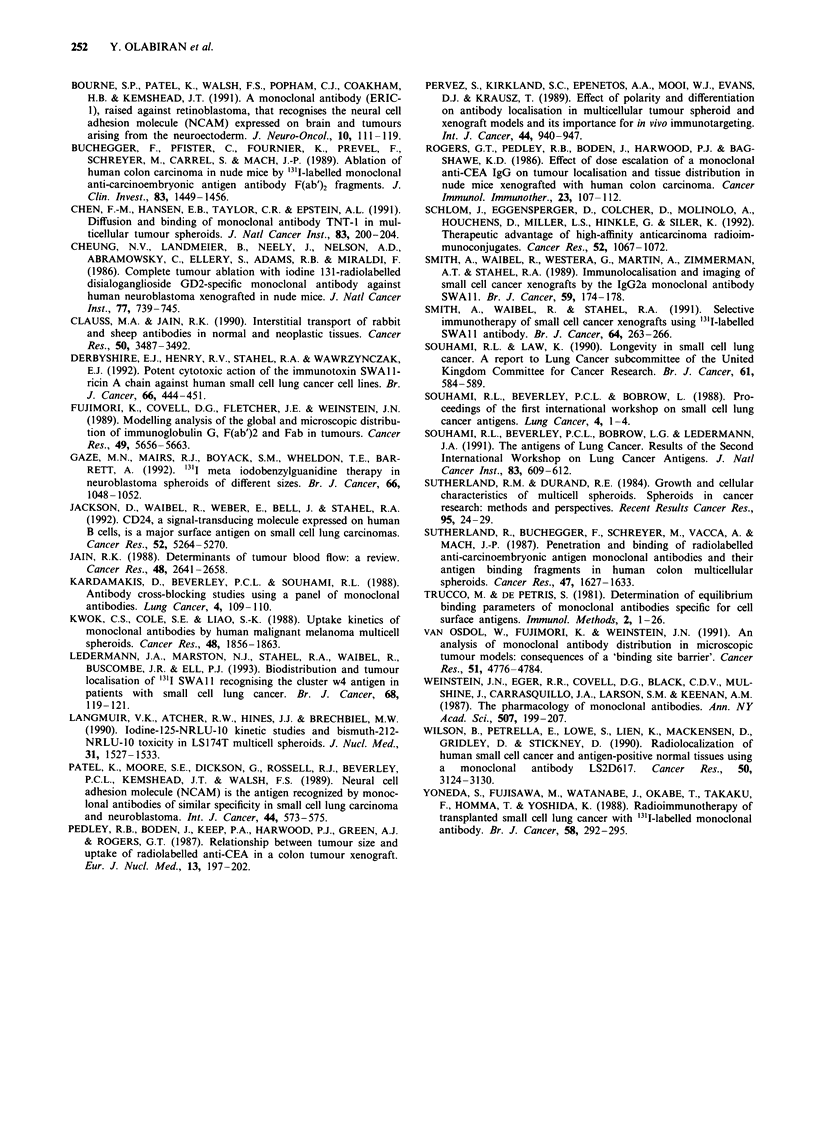# The selection of antibodies for targeted therapy of small-cell lung cancer (SCLC) using a human tumour spheroid model to compare the uptake of cluster 1 and cluster w4 antibodies.

**DOI:** 10.1038/bjc.1994.47

**Published:** 1994-02

**Authors:** Y. Olabiran, J. A. Ledermann, N. J. Marston, G. M. Boxer, R. Hicks, R. L. Souhami, S. G. Spiro, R. A. Stahel

**Affiliations:** Department of Oncology, University College London Medical School, UK.

## Abstract

**Images:**


Br. J. Cancer (1994), 69, 247-252

© Macmillan Press Ltd., 1994

### The selection of antibodies for targeted therapy of small-cell lung cancer (SCLC) using a human tumour spheroid model to compare the uptake of cluster 1 and cluster w4 antibodies

Y. Olabiran[1], J.A. Ledermann[1], N.J. Marston[1], G.M. Boxer[2], R. Hicks[3], R.L. Souhami[1], S.G. Spiro[4] & R.A. Stahel[5]

*[1]Department of Oncology, University College London Medical School, London W1P 8BT, UK; [2]Department of Clinical Oncology, Royal Free Hospital School of Medicine, London NW3 2PF, UK; [3]ICRF Human Tumour Immunology Unit, University College London Medical School, London W1P 8BT, UK; [4]Royal Brompton Hospital, London SW3, UK; [5]Department of Oncology, University of Zurich, Switzerland.*

**Summary** Spheroids of a small-cell lung cancer (SCLC) cell line POC were used to evaluate the uptake and penetration of two antibodies recognising different SCLC antigens. Spheroids approximately 300–400 μm in diameter were incubated with 1 μg ml$^{-1}$ $^{125}$I-labelled NY.3D11, an antibody which reacts with the cluster 1 group antigen (neural cell adhesion molecule; NCAM) and [$^{125}$I]SWA11, which binds to the cluster w4 antigen. The rate of uptake of both antibodies was similar; an initially rapid phase was seen during the first 8 h and maximum uptake occurred by 24 h. The mean uptake per spheroid at 24 h was 0.97 ng for [$^{125}$I]NY.3D11 and 0.45 ng for [$^{125}$I]SWA11. An objective measurement of antibody penetration into spheroids was developed using a computerised image analysis of immunostained sections of spheroids. The concentration of antibody and incubation times were varied. Both antibodies penetrated the spheroids to a depth of 50 μm after 30 min. This increased to about 100 μm after 4 h incubation with 1 or 100 μg ml$^{-1}$ SWA11. The results with 1 μg ml$^{-1}$ NY.3D11 were similar, but in the presence of 100 μg ml$^{-1}$ NY.3D11 penetration into the spheroid was deep and diffuse. These results demonstrate a major concentration-dependent difference in the uptake and penetration of cluster 1 and cluster w4 antibodies in this spheroid model and they have implications for the selection of antibodies for targeted therapy of SCLC.

Successful therapy with antibody conjugates is most likely to be achieved in tumours in which the burden of cells is low and tumour foci are small. Studies in animals with human tumour xenografts have demonstrated that the most favourable anti-tumour antibody uptake ratio is seen in small tumours (Rogers *et al.*, 1986; Pedley *et al.*, 1987), and this probably accounts for the good results of radioimmunotherapy in these animals (Cheung *et al.*, 1986; Buchegger *et al.*, 1989; Smith *et al.*, 1991). In man radiolabelled antibodies have usually been given to treat large tumours, and although responses occur they are rarely sustained. It is likely that the best results of radioimmunotherapy in man will occur when treatment is given as an adjuvant to eradicate persistent micrometastases.

Small-cell lung cancer (SCLC) is a tumour ideally suited to adjuvant antibody-targeted therapy as about half the patients treated with current chemotherapy and radiation protocols enter a complete remission. However, subclinical disease remains as more than 95% of these patients eventually die of their disease (Souhami & Law, 1990). Antibody-targeted radiotherapy could be given to patients in complete remission as at this time individual tumour foci may contain about $10^9$ cells, i.e. 1 cm$^3$ or less. The success of this approach will depend on delivering sufficient antibody to residual tumour deposits and on choosing a radiopharmaceutical with appropriate characteristics for the size of the tumour (Gaze *et al.*, 1992).

There are many antibodies that react with SCLC antigens and that could be considered as suitable agents for targeted therapy. Several of these have been grouped into clusters defined by two international workshops on the basis of a common pattern of reactivity (Souhami *et al.*, 1988, 1991). Radiolabelled antibodies against clusters 1 and w4 have been shown to localise SCLC growing as a xenograft in 'nude mice' (Yoneda *et al.*, 1988; Smith *et al.*, 1989; Wilson *et al.*, 1990; Boerman *et al.*, 1991), and clinical studies to examine their biodistribution and ability to localise in tumours are in progress.

Studies comparing the uptake of different antibodies to SCLC can be performed in mice with xenograft tumours, but it is uncertain whether the results predict the behaviour of such antibodies in man. Studies comparing the uptake of antibodies by tumours are not easily performed in man as it is difficult to obtain tissue. Multicellular tumour spheroids represent an intermediate level of complexity between single cells *in vitro* and solid tumours *in vivo*. They have some of the characteristics of human micrometastases (Sutherland & Durand, 1984) and they have been used as a model to compare the microdistribution of different anti-tumour antibodies (Sutherland *et al.*, 1987) and to evaluate targeted radiotherapy (Gaze *et al.*, 1992).

We have used a human SCLC spheroid model to compare the behaviour of two antibodies that are potential vehicles for antibody-directed radiotherapy of SCLC. One antibody is directed against the cluster 1 antigen, the neural cell adhesion molecule (NCAM) (Patel *et al.*, 1989) expressed on SCLC, and the other is against the cluster w4 antigen (Smith *et al.*, 1989), which has recently been shown to be identical to a leucocyte activation antigen CD24 (Jackson *et al.*, 1992).

### Materials and methods

### *Cells and spheroid culture*

The POC cell line obtained from P. Twentyman (MRC, Cambridge, UK) and the UCH10 cell line (Kardamakis *et al.*, 1988) were used. The SCLC cell lines POC and UCH10 were maintained in RPMI-1640 containing 10% FCS (Gibco Laboratories) and 2 mM glutamine at 37°C in a 5% carbon dioxide incubator. Spheroids were initiated by inoculating $5 \times 10^4$ cells into a 75 ml tissue culture flask containing 50 ml of medium. POC cells grew spontaneously as spheroids in flat-bottomed tissue culture flasks. The size of spheroids was measured using a calibrated ocular eyepiece on a phase-

Correspondence: J.A. Ledermann, Department of Oncology, University College London Medical School, 91 Riding House St, London W1P 8BT, UK.
Received 14 December 1992; and in revised form 28 September 1993.

contrast microscope. Spheroids were harvested usually 7–10 days after inoculation, when their diameter was approximately 300–400 μm. They were disaggregated either mechanically using a pipette or enzymatically using trypsin in order to obtain single-cell suspensions for flow cytometry.

*Monoclonal antibodies*

The monoclonal antibody NY.3D11 was raised following immunisation of RBS/DNJ mice (Robertsonian 8:12 translocation) with live UCH10 cells. Spleen cells from immune animals were fused with the Fox NY, NS-1 variant myeloma line. NY.3D11 was selected using an indirect immunofluorescence assay which detected antibodies that bound to a mouse fibroblast cell line transfected with the human muscle NCAM gene (a gift from F.S. Walsh, Guy's Hospital, London, UK). SWA11 is an IgG2a antibody that recognises the cluster w4 antigen (Smith *et al.*, 1989). Antibodies were purified from tissue culture supernatant by protein A–Sepharose chromatography. Two anti-NCAMs, ERIC-1, an IgG1 (Bourne *et al.*, 1991), and an IgG2a antibody (both supplied by J. Kemshead, ICRF, Frenchay Hospital, Bristol, UK) were used as positive controls. A locally produced IgG1 antibody, QS4120, an anti-human CD4 (P. Beverley, personal communication) was used as a negative control antibody.

Antibodies were radiolabelled with $^{125}$I by the iodogen method with a specific activity of approximately 1 mCi mg$^{-1}$. Antibody immunoreactivity was determined using a cell-binding assay (Trucco & de Petris, 1981). A fixed amount of antibody (approximately 16,500 c.p.m.) was added to a range of UCH10 cells ($10^7$ to $6.25 \times 10^6$) for 2 h at 4°C. After washing bound counts were measured and the number of unbound counts was plotted (*y*-axis) against the reciprocal cell number. The difference between input and unbound counts at the intercept on the *y*-axis gives the theoretical immunoreactive fraction at an infinite excess of antigen. Antibody affinity ($K$) and binding sites ($n$) were determined as described by Trucco and de Petris (1981). Serial twofold dilutions of radiolabelled antibody were added to $10^6$ UCH10 cells in minimum essential medium (MEM) and 2% fetal calf serum (FCS) and left for 2 h on ice. After washing bound counts were measured. The results were expressed as a plot of $r$ against $r/A-X$, where $r$ is the number of antibody molecules bound per cell and $A-X$ is the free antibody expressed as c.p.m.

*Binding of antibodies to POC cells*

An indirect immunofluorescence assay was used to establish binding of all three antibodies to POC cells. A single-cell suspension was prepared and 50 μl of a 10 μg ml$^{-1}$ solution of NY.3D11, ERIC 1 or SWA11 in MEM with 2% FCS was added. Antibodies were incubated with approximately $2 \times 10^5$ cells for 1 h on ice. Following washing fluorescein-conjugated anti-mouse immunoglobulin was added for 45 min on ice. After further washing cells were analysed on a fluorescence-activated cell sorter (FACScan, Becton Dickinson).

*Uptake of radiolabelled antibodies by spheroids*

Radiolabelled antibody, diluted to 1 μg ml$^{-1}$ in MEM containing 2% FCS, was incubated with ten spheroids in 1.5 ml Eppendorf tubes for various times at 37°C. Tubes were agitated periodically and supernatant was removed and spheroids were gently washed with buffer. Experiments were performed in duplicate and the results were expressed as the mean quantity of antibody bound per ten spheroids.

*Penetration of antibodies into spheroids*

Spheroids were incubated with 1 ml of antibody at either 1 or 100 μg ml$^{-1}$ in RPMI containing 2% FCS for 30 min or 4 h. The supernatant medium was carefully removed with a pipette and a few drops of cryo-embedding medium, OCT (Miles), was introduced into the tube held at an angle. A small pipette was used to gently agitate the spheroids and lift them from the bottom of the tube so that the embedding medium could penetrate around them. The whole sealed tube was immersed in liquid nitrogen. The conical-shaped block was mounted on a metal cyrostat chuck at −30°C and 7 μm sections were cut through the tip of the block.

*Immunohistochemical localisation of antibody*

Sections were fixed in 10% buffered formalin for 5 min and bound antibody was localised using an avidin–biotin– peroxidase technique. Briefly, sections were incubated with biotinylated horse anti-mouse immunoglobulin for 30 min, washed in Tris-buffered saline and then incubated with avidin–biotin–peroxidase complexes reagent (Vector Laboratories, UK) for 50 min. Sites of antibody binding were demonstrated following addition of substrate, diaminobenzidine tetrahydrochloride. Staining for the cluster 1 or w4 antigen using SWA11 or NY.3D11, or for the presence of antibody following incubation, was performed on serial sections of spheroid with approximately equivalent diameters. An image analyser that used an 8-bit greyscale program, 'Image', on a Macintosh computer was used to measure the depth of penetration of antibodies. At least two sets of experiments were performed for each antibody.

### Results

*Uptake of cluster 1 and w4 antibodies in SCLC spheroids*

The antibody NY.3D11 binds to human NCAM expressed on the surface of cells. In the experiment shown in Figure 1 the antibody was incubated with D243, a mouse fibroblast cell line that had been transfected with cDNA encoding the 140 kDa isoform of human skeletal muscle NCAM. NCAM is expressed on the surface of these cells and the fluorescence histogram (Figure 1a and b) shows binding of NY.3D11 to D243 but not to the parent mouse fibroblast line. Both NY.3D11 and SWA11 bound to the two SCLC lines, POC and UCH10 cells (Figure 1c and d).

POC spheroids with a diameter of 300–400 μm contained about $10^4$ cells. The proporton of dividing cells was not determined, but visible signs of necrosis were not apparent until the diameter exceeded 500 μm. The binding affinity of NY.3D11 and SWA11 for UCH10 cells is shown in Figure 2. The immunoreactive fraction determined by the method of Trucco and de Petris (1981) was usually 50–60% of the labelled preparation. Preliminary separate experiments with [$^{125}$I]SWA11 and [$^{125}$I]NY.3D11 had shown that the affinity of the two antibodies for their antigen was similar and approximately $10^8$ M$^{-1}$. In a paired experiment the affinity of the two antibodies for UCH10 cells was $2.3 \times 10^8$ M$^{-1}$ for NY.3D11 and $1.1 \times 10^8$ M$^{-1}$ for SWA11. There were approximately $4.4 \times 10^5$ binding sites per cell for NY.3D11 and $2.3 \times 10^6$ sites for SWA11.

The rates of uptake of the cluster 1 and w4 antibodies by POC spheroids were similar. During the first 8 h of incubation there was a rapid increase in uptake of [$^{125}$I]NY.3D11 and [$^{125}$I]SWA11 antibodies, and this was followed by a slower accumulation thereafter. No significant increase in uptake of antibody was seen beyond 24 h (Figure 3). The mean absolute uptake of [$^{125}$I]NY.3D11 was 0.44 ng per spheroid at 4 h, increasing to 0.97 ng by 24 h. In contrast, the mean absolute uptake of [$^{125}$I]SWA11 was 0.13 ng at 4 h and 0.45 ng by 24 h. The uptake kinetics of another anti-NCAM, [$^{125}$I]ERIC-1, was similar to that of [$^{125}$I]NY.3D11, and the mean absolute uptake of this antibody was 0.37 ng and 0.83 ng per spheroid at 4 and 24 h respectively. The uptake of [$^{125}$I]4120 at the same time points was 0.14 ng and 0.16 ng.

**Figure 1** Fluorescence histogram demonstrating **a**, The binding of NY.3D11 to D243 (NCAM transfectant); and **b**, The parent mouse fibroblast (L cells). Binding of NY.3D11 and SWA11 to UCH10 and POC cells is shown in **c** and **d**. Solid lines represent fluorescence following incubation of FITC-labelled anti-mouse immunoglobulin only (control).

*Penetration of antibodies into SCLC spheroids*

Computerised image analysis was used to provide an objective measurement of the depth of penetration of antibodies. It showed that differences in the penetration of the two antibodies into spheroids were not significantly different in the presence of $1\ \mu g\ ml^{-1}$ antibody but were strikingly different in the presence of $100\ \mu g\ ml^{-1}$ (Figure 4). The distribution of NY.3D11 across the spheroid section was fairly uniform, but deposition of SWA11 was seen mainly in a rim extending no more than $100\ \mu m$ from the surface of the spheroid. These differences are clearly seen in the photomicrographs shown in Figure 5. Following 30 min incubation NY.3D11 and SWA11 had penetrated to a depth of about 3–4 cell layers ($50\ \mu m$) from the surface of the spheroid. The intensity of staining of SWA11 at 4 h is greater than NY.3D11, but the latter has penetrated more deeply and diffusely into the spheroid. The antibodies appeared to be localised around the surface of the cells. No uptake of the control antibody, QS4120, was seen at 4 h.

Direct incubation of tissue sections with antibody showed a homogeneous distribution of cluster 1 and cluster w4 antigens (Figure 4). Antigen was not detected in areas of central necrosis seen in some spheroids larger than $400\ \mu m$ in diameter. A high intensity of staining was seen at the rim of the spheroids stained for antigen. This peak is unlikely to be an artefact due to high concentrations of antibody in the surrounding medium. It could have been due to tissue shrinkage during penetration of the spheroid or to a true increase in the concentration of antigen at the surface of the spheroid.

**Figure 2** Scatchard plot showing the binding of **a**, [$^{125}$I]SWA11; and **b**, [$^{125}$I]NY.3D11 to UCH10 cells. For SWA11 the affinity ($K$) = $1.1 \times 10^8\ M^{-1}$ and the number of binding sites per cell ($n$) = $2.3 \times 10^6$. For NY.3D11, $K = 2.3 \times 10^8\ M^{-1}$ and $n = 4.4 \times 10^5$.

### Discussion

The purpose of this study was to compare the uptake and penetration of two distinct antibodies to SCLC antigens in SCLC tumours. Human tumour spheroids were used as they have some features in common with micrometastatic SCLC. Both radiolabelled cluster 1 and cluster w4 antibodies have been shown to have therapeutic activity in animals with SCLC xenograft tumours (Yoneda *et al.*, 1988; Smith *et al.*,

**Figure 3** Uptake of $^{125}$I-labelled antibodies to cluster 1 and w4 antigens and control antibody (1 $\mu$g ml$^{-1}$) in POC spheroids. Mean uptake and standard deviation are shown.

1991) and cluster 1 and w4 antigens are commonly found in tumours of patients (Souhami et al., 1991). The patterns of uptake of NY.3D11 and SWA11 in POC spheroids were similar. A rapid phase of accumulation during the first 8 h was followed by only a small increase in uptake over the next 16 h. The decrease in uptake of radiolabelled antibody after 8 h was not due to exhaustion of the supply of antibody as only a small fraction of antibody in the incubation medium bound to spheroids. The results are similar to those reported in studies of human melanoma, colon and ovarian carcinoma spheroids (Kwok et al., 1988; Langmuir et al., 1990; Bardiès et al., 1992). However, a clear difference in the absolute amount of anti-SCLC antibody accumulating in the spheroids was seen. Following incubation with 1 $\mu$g ml$^{-1}$ antibody for 24 h each spheroid contained about 1 ng of [$^{125}$I]NY.3D11. This was approximately twice the amount of [$^{125}$I]SWA11. Similar results were obtained with the anti-NCAM, ERIC-1, which suggests that the different behaviour was due to interaction of the antibody with its antigen. It is possible that the $\gamma_{2a}$ subclass of SWA11 behaved differently from the $\gamma_1$ subclass of NY.3D11. However, when we used an IgG2a cluster 1 antibody we did not see any difference in penetration into the spheroid compared with the IgG1 cluster 1 antibody (data not shown). Antibody uptake in tumour spheroid models can be improved by greatly increasing the concentration of the incubating antibody (Kwok et al., 1988; Langmuir et al., 1990; Bardiès et al., 1992). Studies were performed with 1 $\mu$g ml$^{-1}$ antibody, as this is a concentration that can be realistically maintained in the blood of patients for several hours. Accumulation of [$^{125}$I]NY.3D11 at this dose was greater than that of [$^{125}$I]SWA11, but the difference was not such that any change in the depth of penetration of the two antibodies was detectable by immunohistochemistry. A major difference in the depth of penetration of the two antibodies was seen only with 100 $\mu$g ml$^{-1}$ incubating antibody. While it is possible to achieve high peak levels of antibody in blood, it is not clear whether high levels can be sustained for a sufficient time to alter the depth of penetration of an antibody.

Most previous studies with radiolabelled antibodies have shown that much of the radioactivity resides on the surface or outer layers of spheroids expressing membrane antigens (Sutherland et al., 1987; Pervez et al., 1989; Langmuir et al., 1990; Chen et al., 1991). The inability of antibodies to penetrate deeply into a tumour mass reduces the efficacy of radioimmunoconjugate therapy (Bardiès et al., 1992). Much work has been devoted to studying factors that affect the uptake and penetration of anti-tumour antibodies in tumours, as they have an important bearing on the success of antibody-targeted therapy. The size of the tumour (Pedley et al., 1987), local interstitial pressures (Jain, 1988) and size of the immunoglobulin molecule (Sutherland et al., 1987) are all likely to be influential factors. Furthermore, antigens may not be homogeneously distributed on the surface membrane.

**Figure 4** **a** and **b**, Image analysis of POC spheroid sections showing the presence of cluster 1 or w4 antigen following incubation with NY.3D11 or SWA11 (1 $\mu$g ml$^{-1}$). The relative optical density following incubation with control antibody QS4120 was between 80 and 90. **c** and **d**, A comparison of the depth of penetration of 100 $\mu$g ml$^{-1}$ NY.3D11 and SWA11 after incubation for 4 h.

**Figure 5** Immunohistochemical staining demonstrating the uptake of SWA11 after **a**, 30 min incubation with POC spheroids; and **b**, 4 h incubation. Uptake of NY.3D11 at 4 h is shown in **c**. Antibody concentration was 100 μg ml$^{-1}$. Magnification × 200.

For example, Pervez *et al.* (1989) have shown that in spheroid models some antigens are expressed preferentially on the membrane of the outer surface of the spheroid. Nuclear antigens are exposed by necrotic cells, and this results in preferential binding of antibody at the centre of the spheroid (Chen *et al.*, 1991). Antibody uptake and penetration into a tumour may also depend on antibody affinity (Weinstein *et al.*, 1987; Schlom *et al.*, 1992) and physicochemical properties of antibodies (Clauss & Jain, 1990).

In the POC spheroid model computerised image analysis of immunohistochemically stained tissue sections suggested that the higher uptake of cluster 1 antibody, NY.3D11, was due to its deeper penetration into the spheroid. While we did not compare the absolute uptake of NY.3D11 and SWA11 into spheroids at high and low concentrations, we have shown that the depth of penetration of SWA11 cannot be increased to more than about 100 μm from the surface. These findings suggest there is a fundamental difference in the behaviour of these two antibodies in this spheroid model system.

The difference in penetration of the antibodies was not related to the distribution of cluster 1 and w4 antigens, which was shown by immunohistochemistry to be fairly uniform and on the surface membrane of cells. However, SWA11 is internalised by cells (Derbyshire *et al.*, 1992), and this may retard its penetration through spheroids. There is no evidence that the cluster 1 antibodies are internalised (R.L. Souhami, unpublished observations). Alternatively, differences in the depth of penetration of antibodies into spheroids could be related to antibody affinity. It has been suggested that the interaction of high-affinity antibodies at the surface of a tumour prevents its penetration (Fujimori *et al.*, 1989; van Osdol *et al.*, 1991). We were unable to measure reproducibly the affinity of the antibodies on POC cells, as it was difficult to prepare a single-cell suspension that was not heavily contaminated by dead cells which accumulated radiolabelled antibody non-specifically. There was little difference in the affinity of SWA11 and NY.3D11 on UCH10 cells which grow as a cell suspension. It is unlikely that the affinity of these antibodies for their antigen would be significantly different on POC cells and we therefore believe that the differences in the penetration of these antibodies into spheroids are unlikely to be related to their affinity for the antigen. However, there were approximately five times as many antigen binding sites for SWA11 on UCH10 compared with NY.3D11. Cell-binding studies by FACS analysis showed that the mean fluorescence from SWA11 binding to UCH10 and POC was greater than from NY.3D11 (Figure 1), suggesting that there were more antigen binding sites for SWA11 on both cell lines. If the antigen densities on POC and UCH10 are similar then one could envisage that a high density of antigen might lead to a deposition of SWA11 near the surface of the spheroid, forming a barrier which might prevent further penetration of antibody. The lower density of cluster 1 antigen might permit a more diffuse penetration of antibody through the spheroid.

Differences in the behaviour of antibodies in the tumour environment are difficult to measure in patients, and in particular in SCLC, in which the aim of treatment is to eradicate small-volume residual disease. The spheroid model of SCLC has been shown to be a useful method of studying differences in the pattern of uptake of cluster 1 and w4 antibodies. The results may have implications for the choice of antibody and conjugate most likely to be effective in therapy of SCLC. However, selection of the most suitable radiopharmaceutical will also depend upon the results of clinical studies that examine biodistribution and tumour localisation of these two antibodies. We have recently shown that radiolabelled SWA11 localises in tumours, but it also accumulates in areas rich in granulocytes, making it unsuitable for targeted therapy of SCLC (Ledermann *et al.*, 1993). A study of the biodistribution of radiolabelled NY.3D11 in patients is to start shortly.

Dr Olabiran was supported by the Ernest Ringveldt Bequest from the Royal Brompton, National Heart and Lung Hospital, London. We would like to thank Mr F. Moll for assistance in preparing the photomicrographs.

**References**

BARDIÈS, M., THEDREZ, P., GESTIN, J.-F., MARCILLE, B.-M., GUERREAU, D., FAIVRE-CHAUVET, A., MAHÉ, M., SAI-MAUREL, C. & CHATAL, J.-F. (1992). Use of multi-cell spheroids of ovarian carcinoma as an intraperitoneal radio-immunotherapy model: uptake, retention kinetics and dosimetric evaluation. *Int. J. Cancer*, **50**, 984–991.

BOERMAN, O., MIJNHEERE, E., BROERS, J., VOOIJS, G. & RAMAEKERS, F. (1991). Biodistribution of a monoclonal antibody (RNL-1) against the neural cell adhesion molecule (NCAM) in athymic mice bearing human small-cell lung cancer xenografts. *Int. J. Cancer*, **48**, 457–462.

BOURNE, S.P., PATEL, K., WALSH, F.S., POPHAM, C.J., COAKHAM, H.B. & KEMSHEAD, J.T. (1991). A monoclonal antibody (ERIC-1), raised against retinoblastoma, that recognises the neural cell adhesion molecule (NCAM) expressed on brain and tumours arising from the neuroectoderm. *J. Neuro-Oncol.*, **10**, 111–119.

BUCHEGGER, F., PFISTER, C., FOURNIER, K., PREVEL, F., SCHREYER, M., CARREL, S. & MACH, J.-P. (1989). Ablation of human colon carcinoma in nude mice by $^{131}$I-labelled monoclonal anti-carcinoembryonic antigen antibody F(ab')$_2$ fragments. *J. Clin. Invest.*, **83**, 1449–1456.

CHEN, F.-M., HANSEN, E.B., TAYLOR, C.R. & EPSTEIN, A.L. (1991). Diffusion and binding of monoclonal antibody TNT-1 in multicellular tumour spheroids. *J. Natl Cancer Inst.*, **83**, 200–204.

CHEUNG, N.V., LANDMEIER, B., NEELY, J., NELSON, A.D., ABRAMOWSKY, C., ELLERY, S., ADAMS, R.B. & MIRALDI, F. (1986). Complete tumour ablation with iodine 131-radiolabelled disialoganglioside GD2-specific monoclonal antibody against human neuroblastoma xenografted in nude mice. *J. Natl Cancer Inst.*, **77**, 739–745.

CLAUSS, M.A. & JAIN, R.K. (1990). Interstitial transport of rabbit and sheep antibodies in normal and neoplastic tissues. *Cancer Res.*, **50**, 3487–3492.

DERBYSHIRE, E.J., HENRY, R.V., STAHEL, R.A. & WAWRZYNCZAK, E.J. (1992). Potent cytotoxic action of the immunotoxin SWA11-ricin A chain against human small cell lung cancer cell lines. *Br. J. Cancer*, **66**, 444–451.

FUJIMORI, K., COVELL, D.G., FLETCHER, J.E. & WEINSTEIN, J.N. (1989). Modelling analysis of the global and microscopic distribution of immunoglobulin G, F(ab')2 and Fab in tumours. *Cancer Res.*, **49**, 5656–5663.

GAZE, M.N., MAIRS, R.J., BOYACK, S.M., WHELDON, T.E., BARRETT, A. (1992). $^{131}$I meta iodobenzylguanidine therapy in neuroblastoma spheroids of different sizes. *Br. J. Cancer*, **66**, 1048–1052.

JACKSON, D., WAIBEL, R., WEBER, E., BELL, J. & STAHEL, R.A. (1992). CD24, a signal-transducing molecule expressed on human B cells, is a major surface antigen on small cell lung carcinomas. *Cancer Res.*, **52**, 5264–5270.

JAIN, R.K. (1988). Determinants of tumour blood flow: a review. *Cancer Res.*, **48**, 2641–2658.

KARDAMAKIS, D., BEVERLEY, P.C.L. & SOUHAMI, R.L. (1988). Antibody cross-blocking studies using a panel of monoclonal antibodies. *Lung Cancer*, **4**, 109–110.

KWOK, C.S., COLE, S.E. & LIAO, S.-K. (1988). Uptake kinetics of monoclonal antibodies by human malignant melanoma multicell spheroids. *Cancer Res.*, **48**, 1856–1863.

LEDERMANN, J.A., MARSTON, N.J., STAHEL, R.A., WAIBEL, R., BUSCOMBE, J.R. & ELL, P.J. (1993). Biodistribution and tumour localisation of $^{131}$I SWA11 recognising the cluster w4 antigen in patients with small cell lung cancer. *Br. J. Cancer*, **68**, 119–121.

LANGMUIR, V.K., ATCHER, R.W., HINES, J.J. & BRECHBIEL, M.W. (1990). Iodine-125-NRLU-10 kinetic studies and bismuth-212-NRLU-10 toxicity in LS174T multicell spheroids. *J. Nucl. Med.*, **31**, 1527–1533.

PATEL, K., MOORE, S.E., DICKSON, G., ROSSELL, R.J., BEVERLEY, P.C.L., KEMSHEAD, J.T. & WALSH, F.S. (1989). Neural cell adhesion molecule (NCAM) is the antigen recognized by monoclonal antibodies of similar specificity in small cell lung carcinoma and neuroblastoma. *Int. J. Cancer*, **44**, 573–575.

PEDLEY, R.B., BODEN, J., KEEP, P.A., HARWOOD, P.J., GREEN, A.J. & ROGERS, G.T. (1987). Relationship between tumour size and uptake of radiolabelled anti-CEA in a colon tumour xenograft. *Eur. J. Nucl. Med.*, **13**, 197–202.

PERVEZ, S., KIRKLAND, S.C., EPENETOS, A.A., MOOI, W.J., EVANS, D.J. & KRAUSZ, T. (1989). Effect of polarity and differentiation on antibody localisation in multicellular tumour spheroid and xenograft models and its importance for *in vivo* immunotargeting. *Int. J. Cancer*, **44**, 940–947.

ROGERS, G.T., PEDLEY, R.B., BODEN, J., HARWOOD, P.J. & BAGSHAWE, K.D. (1986). Effect of dose escalation of a monoclonal anti-CEA IgG on tumour localisation and tissue distribution in nude mice xenografted with human colon carcinoma. *Cancer Immunol. Immunother.*, **23**, 107–112.

SCHLOM, J., EGGENSPERGER, D., COLCHER, D., MOLINOLO, A., HOUCHENS, D., MILLER, L.S., HINKLE, G. & SILER, K. (1992). Therapeutic advantage of high-affinity anticarcinoma radioimmunoconjugates. *Cancer Res.*, **52**, 1067–1072.

SMITH, A., WAIBEL, R., WESTERA, G., MARTIN, A., ZIMMERMAN, A.T. & STAHEL, R.A. (1989). Immunolocalisation and imaging of small cell cancer xenografts by the IgG2a monoclonal antibody SWA11. *Br. J. Cancer*, **59**, 174–178.

SMITH, A., WAIBEL, R. & STAHEL, R.A. (1991). Selective immunotherapy of small cell cancer xenografts using $^{131}$I-labelled SWA11 antibody. *Br. J. Cancer*, **64**, 263–266.

SOUHAMI, R.L. & LAW, K. (1990). Longevity in small cell lung cancer. A report to Lung Cancer subcommittee of the United Kingdom Committee for Cancer Research. *Br. J. Cancer*, **61**, 584–589.

SOUHAMI, R.L., BEVERLEY, P.C.L. & BOBROW, L. (1988). Proceedings of the first international workshop on small cell lung cancer antigens. *Lung Cancer*, **4**, 1–4.

SOUHAMI, R.L., BEVERLEY, P.C.L., BOBROW, L.G. & LEDERMANN, J.A. (1991). The antigens of Lung Cancer. Results of the Second International Workshop on Lung Cancer Antigens. *J. Natl Cancer Inst.*, **83**, 609–612.

SUTHERLAND, R.M. & DURAND, R.E. (1984). Growth and cellular characteristics of multicell spheroids. Spheroids in cancer research: methods and perspectives. *Recent Results Cancer Res.*, **95**, 24–29.

SUTHERLAND, R., BUCHEGGER, F., SCHREYER, M., VACCA, A. & MACH, J.-P. (1987). Penetration and binding of radiolabelled anti-carcinoembryonic antigen monoclonal antibodies and their antigen binding fragments in human colon multicellular spheroids. *Cancer Res.*, **47**, 1627–1633.

TRUCCO, M. & DE PETRIS, S. (1981). Determination of equilibrium binding parameters of monoclonal antibodies specific for cell surface antigens. *Immunol. Methods*, **2**, 1–26.

VAN OSDOL, W., FUJIMORI, K. & WEINSTEIN, J.N. (1991). An analysis of monoclonal antibody distribution in microscopic tumour models: consequences of a 'binding site barrier'. *Cancer Res.*, **51**, 4776–4784.

WEINSTEIN, J.N., EGER, R.R., COVELL, D.G., BLACK, C.D.V., MULSHINE, J., CARRASQUILLO, J.A., LARSON, S.M. & KEENAN, A.M. (1987). The pharmacology of monoclonal antibodies. *Ann. NY Acad. Sci.*, **507**, 199–207.

WILSON, B., PETRELLA, E., LOWE, S., LIEN, K., MACKENSEN, D., GRIDLEY, D. & STICKNEY, D. (1990). Radiolocalization of human small cell cancer and antigen-positive normal tissues using a monoclonal antibody LS2D617. *Cancer Res.*, **50**, 3124–3130.

YONEDA, S., FUJISAWA, M., WATANABE, J., OKABE, T., TAKAKU, F., HOMMA, T. & YOSHIDA, K. (1988). Radioimmunotherapy of transplanted small cell lung cancer with $^{131}$I-labelled monoclonal antibody. *Br. J. Cancer*, **58**, 292–295.